# The Lipid A from the Haloalkaliphilic Bacterium *Salinivibrio sharmensis* Strain BAG^T^

**DOI:** 10.3390/md11010184

**Published:** 2013-01-21

**Authors:** Sara Carillo, Giuseppina Pieretti, Buko Lindner, Ida Romano, Barbara Nicolaus, Rosa Lanzetta, Michelangelo Parrilli, Maria Michela Corsaro

**Affiliations:** 1 Department of Chemical Sciences, University of Naples “Federico II”, Complesso Universitario Monte S. Angelo, Via Cintia 4, 80126 Naples, Italy; E-Mails: sara.carillo2@unina.it (S.C.); gpieretti@unina.it (G.P.); lanzetta@unina.it (R.L.); parrilli@unina.it (M.P.); 2 Division of Immunochemistry, Research Center Borstel, Leibniz-Center for Medicine and Biosciences, Parkallee 10, D-23845 Borstel, Germany; E-Mail: blindner@fz-borstel.de; 3 CNR Institute of Biomolecular Chemistry (ICB-CNR), National Research Council (CNR), Via Campi Flegrei 34, 80078 Pozzuoli, Italy; E-Mails: ida.romano@icb.cnr.it (I.R.); bnicolaus@icb.cnr.it (B.N.)

**Keywords:** endotoxin, lipid A, mass spectrometry, *Salinivibrio sharmensis*, haloalkaliphile

## Abstract

Lipid A is a major constituent of the lipopolysaccharides (or endotoxins), which are complex amphiphilic macromolecules anchored in the outer membrane of Gram-negative bacteria. The glycolipid lipid A is known to possess the minimal chemical structure for LPSs endotoxic activity, able to cause septic shock. Lipid A isolated from extremophiles is interesting, since very few cases of pathogenic bacteria have been found among these microorganisms. In some cases their lipid A has shown to have an antagonist activity, *i.e.*, it is able to interact with the immune system of the host without triggering a proinflammatory response by blocking binding of substances that could elicit such a response. However, the relationship between the structure and the activity of these molecules is far from being completely clear. A deeper knowledge of the lipid A chemical structure can help the understanding of these mechanisms. In this manuscript, we present our work on the complete structural characterization of the lipid A obtained from the lipopolysaccharides (LPS) of the haloalkaliphilic bacterium *Salinivibrio sharmensis*. Lipid A was obtained from the purified LPS by mild acid hydrolysis. The lipid A, which contains different number of fatty acids residues, and its partially deacylated derivatives were completely characterized by means of electrospray ionization Fourier transform ion cyclotron (ESI FT-ICR) mass spectrometry and chemical analysis.

## 1. Introduction

Saline environments, for example deep sea or saline lakes, are the perfect habitat for extremophilic microorganisms such as haloalkaliphiles. In these hard life conditions (high pH values, saturated salt solutions), these bacteria are forced to modify the structure and the composition of their cell; in fact, they undergo strong osmotic stress that is overcome by accumulating osmolytes or by maintaining a cytoplasmic salt concentration (KCl) close to that of the surrounding medium [1,2]. As a consequence, proteins from halophiles are constituted by negatively charged amino acid residues on their surface [3], and membrane phospholipids become more anionic [4,5].

The outer cellular membrane of Gram-negative bacteria is mainly composed of lipopolysaccharides (LPS), amphiphilic macromolecules anchored in the phospholipid bilayer with their lipid part. The LPS amphiphilic nature is defined by their three different covalently linked regions. These are: The hydrophilic polysaccharide portion composed of the *O*-specific antigen, which glycosylates the core oligosaccharide. The latter glycosylates the lipophilic glycolipid portion of lipid A. In particular, the lipid A is an immunomodulatory molecule and is recognized by different classes of receptors on both mammalian and plant cells [6]. It has a conserved architecture consisting of a β-D-GlcN′-(1′→6)-D-GlcN disaccharide (Glc*p*N is 2-amino-2-deoxy-D-glucopyranose) carrying an α-glycosidic-bound phosphate group at position C-1 of the proximal reducing glucosamine residue (Glc*p*N I) and an ester-bound one at position 4′ of the distal, non-reducing end glucosamine residue (Glc*p*N II). Both amino sugar units are substituted with a number of ester- and amide-linked fatty acids. These fatty acids are peculiar because they bear a 3-OH group, which, in turn, can be esterified by other fatty acids residues [7]. The lipid A fraction is intrinsically heterogeneous due to the different degree of phosphorylation and acylation, the distribution and the type of acyl residues. The toxicity of the LPSs depends strongly on the lipid A structure, and is also influenced by the core region [8,9,10].

Among the extremophiles very few cases of pathogenic bacteria have been found. To the best of our knowledge the lipopolysaccharides from the haloalkaliphilic pathogenic bacteria *Halomonas stevensii* were the only ones, which have been characterized [11] so far. In some other cases, endotoxins isolated from extremophilic bacteria have been found to exert an antagonist activity, *i.e.*, they are able to interact with the immune system of the host without triggering a proinflammatory response blocking the binding of substances that could elicit such response [12]. However, the relationship between structure and activity of these molecules is by far not completely clear. A deeper knowledge of the lipid A chemical structure can help the understanding of these mechanisms.

From the saline lake in Ras Mohammed Park (Egypt), a novel haloalkaliphilic, facultative anaerobic and Gram-negative microorganism (designated strain BAG^T^) has recently been recovered. It was identified as a novel species of the *Salinivibrio* genus, and named *Salinivibrio sharmensis* [13].

In this manuscript, we present the complete structural characterization of the lipid A obtained from its LPS. To obtain the lipid A, dried cells were extracted by using the phenol-chloroform-petroleum ether (PCP) method and the LPS was purified and hydrolyzed under mild acidic conditions. The lipid A and its partially deacylated derivatives were completely characterized by means of electrospray ionization Fourier transform ion cyclotron (ESI FT-ICR) mass spectrometry and chemical analysis in order to reveal the nature and the distribution of fatty acids linked to the disaccharidic glucosamine backbone. 

## 2. Results and Discussion

### 2.1. Isolation of Lipid A and Its Compositional Analysis

Dried cells from haloalkaliphilic bacterium *Salinivibrio sharmensis* BAG^T^ were extracted by the PCP method [14]. The obtained lipopolysaccharidic fraction was hydrolyzed under mild acidic condition, with 5% acetic acid. The gas chromatography mass spectrography (GC-MS) analysis of the fatty acids methyl esters derivative, obtained after the LPS methanolysis, showed the presence of 3-hydroxy-dodecanoic [C12:0(3-OH)] and 3-hydroxy-tetradecanoic acids [C14:0(3-OH)], as the most abundant species, together with dodecanoic (C12:0), tetradecanoic (C14:0) and hexadecenoic (C16:1) acids.

### 2.2. ESI FT-ICR Mass Spectrometric Analysis of Lipid A

**Figure 1:**
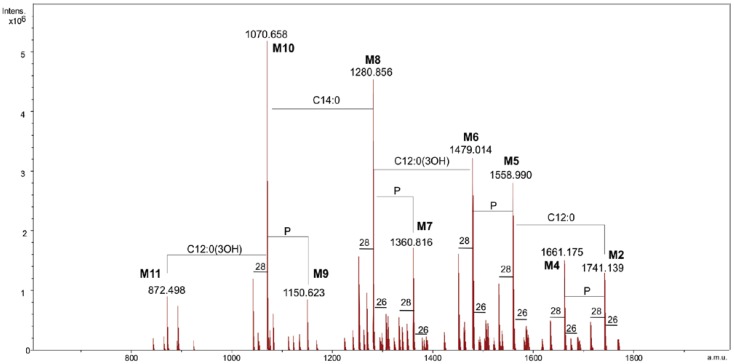
The charge deconvoluted negative ion electrospray ionization Fourier transform ion cyclotron (ESI FT-ICR) mass spectrum of the lipid A from *Salinivibrio sharmensis* strain BAG^T^.

Lipid A was analyzed by ESI FT-ICR mass spectrometry. The charge deconvoluted negative ions ESI FT-ICR mass spectrum showed the presence of nine signals (**M1***–***M9**, Figure 1, Table 1) corresponding to a number of glycoforms differing in their acylation as well as their phosphorylation degree. In particular, they ranged from hexa-acylated to di-acylated glycoforms and, except for **M9**, were mono or bis-phosphorylated. 

**Table 1:** Composition of the main species present in the charge deconvoluted negative ions ESI FT-ICR mass spectrum of the lipid A from *Salinivibrio sharmensis* strain BAG^T^.

Species	M_obs_	M_acc_	Composition
**M1**	1741.14	1741.17	GlcN_2_P_2_[C12:0(3-OH)]_2_[C14:0(3-OH)]_2_(C12:0)(C14:0)
**M2**	1661.18	1661.21	GlcN_2_P[C12:0(3-OH)]_2_[C14:0(3-OH)]_2_(C12:0)(C14:0)
**M3**	1558.99	1559.00	GlcN_2_P_2_[C12:0(3-OH)]_2_[C14:0(3-OH)]_2_(C14:0)
**M4**	1479.01	1479.04	GlcN_2_P[C12:0(3-OH)]_2_[C14:0(3-OH)]_2_(C14:0)
**M5**	1360.82	1360.84	GlcN_2_P_2_[C12:0(3-OH)][C14:0(3-OH)]_2_(C14:0)
**M6**	1280.86	1280.87	GlcN_2_P[C12:0(3-OH)][C14:0(3-OH)]_2_(C14:0)
**M7**	1150.62	1150.64	GlcN_2_P_2_[C12:0(3-OH)][C14:0(3-OH)]_2_
**M8**	1070.66	1070.67	GlcN_2_P[C12:0(3-OH)][C14:0(3-OH)]_2_
**M9**	872.50	872.51	GlcN_2_P[C14:0(3-OH)]_2_

In particular **M1**, **M3**, **M5** and **M7** corresponded to the bis-phosphorylated glycoforms. The following composition was attributed to the species **M1**, with the mass 1741.14 Da, GlcN_2_P_2_[C12:0(3-OH)]_2_[C14:0(3-OH)]_2_(C12:0)(C14:0) (calculated molecular mass: 1741.17 Da). Species **M3** was identified as penta-acylated lipid A since it carries no C12:0 fatty acid, while the **M5** species was missing a C12:0 and 3-hydroxy dodecanoic acid. The tri-acylated species **M7** contained only one 3-hydroxy dodecanoic and two 3-hydroxy tetradecanoic acids. Finally **M9** was the only di-acylated species corresponding to the following composition GlcN_2_P[C14:0(3-OH)]_2_. Less abundant species, due to the replacement of the C14:0 with a C16:1 (+26 u) or a C12:0 (−28 u) fatty acid, were also present. In order to obtain the detailed information on the distribution of fatty acids on the disaccharidic backbone, MS and MS/MS spectra in the positive ion mode were generated.

The FT-ICR-MS (positive ion mode) of lipid A showed the presence of the adduct ion [M + Et_3_N + H]^+^ at *m/z* 1843.31, which corresponded to the hexa-acylated, bis-phosphorylated glycoform (calculated molecular mass: *m/z* 1843.303). The MS/MS spectrum of this species, obtained by infrared multiphoton dissociation (IRMPD) (see experimental section), displayed a B^+^ fragment ion [15,16] at *m/z* 1058.76, to which the following composition was attributed GlcNP[C12:0(3-OH)][C14:0(3-OH)](C12:0)(C14:0) (calculated mass *m/z* 1058.77). These results proved that both the secondary fatty acids (C12:0 and C14:0) were linked to the distal non-reducing end glucosamine.

### 2.3. Analysis of NH_4_OH Product

**Figure 2:**
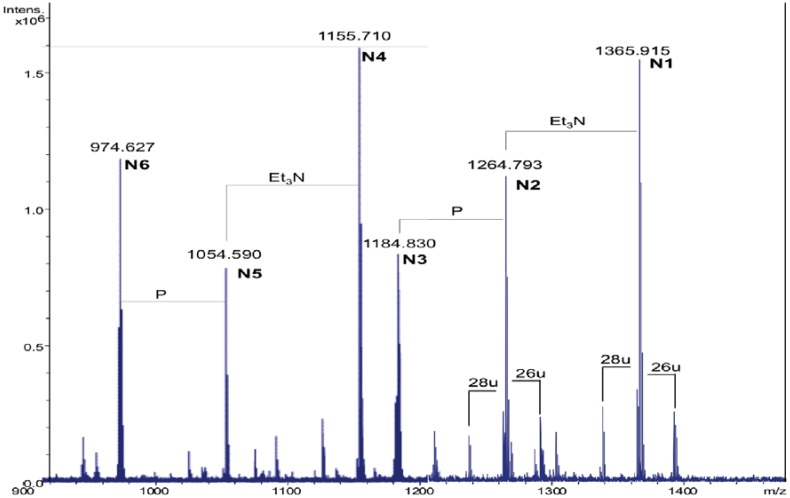
The charge deconvoluted positive ion ESI FT-ICR mass spectrum of the NH_4_OH product from the lipid A of *Salinivibrio sharmensis* strain BAG^T^.

By treating the sample with NH_4_OH [17], the lipid A was deprived of primary *O*-linked fatty acids. The product was analyzed again in the positive ion mode. The mass spectrum revealed the presence of four main signals corresponding to [M + H]^+^ adducts with one or two Et_3_N molecules, followed by the signals of the corresponding monophosphorylated species at 80 u lower (**N1***–***N6**, Figure 2, Table 2).

**Table 2:** Composition of the main species present in the positive ions ESI FT-ICR mass spectrum of the NH_4_OH product of the lipid A from *S. sharmensis* strain BAG^T^.

Species	Observed *m/z*	Calculated *m/z*	Composition
**N1**	1365.91 ^a^	1365.92	GlcN_2_P_2_[C14:0(3-OH)]_2_(C14:0)
**N2**	1264.79 ^b^	1264.80	GlcN_2_P_2_[C14:0(3-OH)]_2_(C14:0)
**N3**	1184.83 ^b^	1184.84	GlcN_2_P[C14:0(3-OH)]_2_(C14:0)
**N4**	1155.71 ^a^	1155.72	GlcN_2_P_2_[C14:0(3-OH)]_2_
**N5**	1054.59 ^b^	1054.60	GlcN_2_P_2_[C14:0(3-OH)]_2_
**N6**	974.63 ^b^	974.64	GlcN_2_P[C14:0(3-OH)]_2_

^a^ This signal corresponds to the adduct [M + 2Et_3_N + H]^+^; ^b^ This signal corresponds to the adduct [M + Et_3_N + H]^+^.

**Figure 3:**
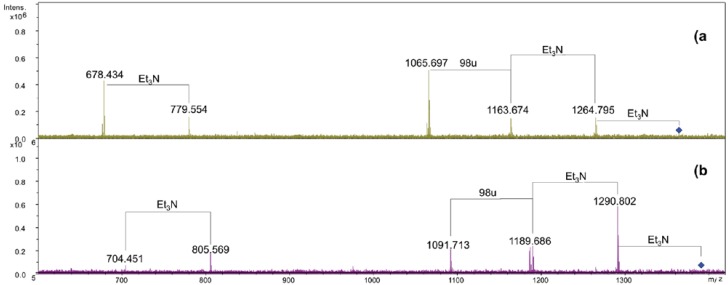
ESI FT-ICR tandem mass spectrum infrared multiphoton dissociation (IRMPD). (**a**) Product ion scan of the isolated precursor ion 1365.9 *m/z*. (**b**) Product ion scan of the isolated precursor ion 1391.9 *m/z*.

The [M + 2Et_3_N + H]^+^ adduct ion at 1365.91 *m/z* (**N1**) was selected for fragmentation. The MS/MS spectrum showed the presence of a B^+^ fragment ion at *m/z* 678.4 (GlcNP[C14:0(3-OH)]C14:0, calculated *m/z* 678.3, Figure 3a), thus revealing that C14:0 was linked as acyloxyamide to the GlcN II. As a consequence, C12:0 resulted to be linked as acyloxyacyl to the GlcN II. In Figure 2, starting from the **N1** signal, a species at 26 u higher masses was found (*m/z* 1391.90), which corresponded to the replacement of the C14:0 with C16:1, as already found.

**Scheme 1:**
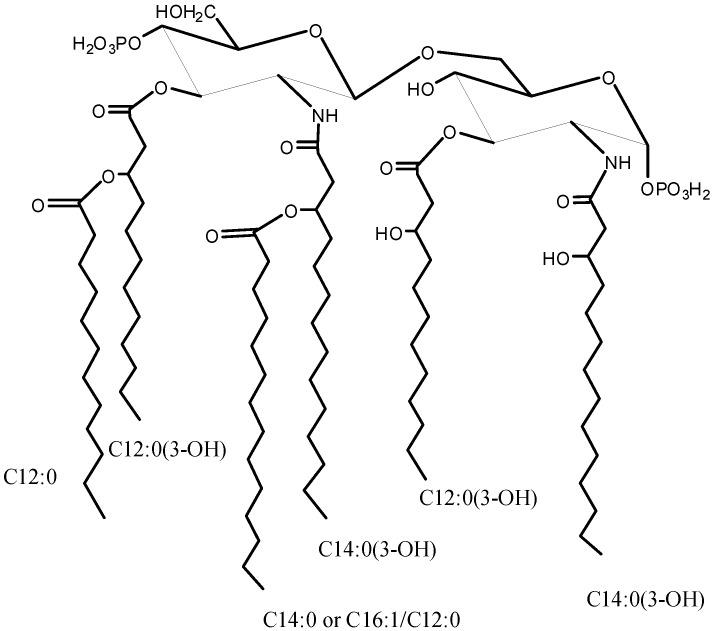
Structure of the Lipid A from *Salinivibrio sharmensis* strain BAG^T^.

The MS/MS spectrum of the signal at *m/z* 1391.9 (Figure 3b) displayed the presence of a B^+^ fragment ion at *m/z* 704.4 confirming that C16:1 occupies the same position of C14:0. The MS/MS spectrum of **N3** showed the presence of a B^+^ fragment ion at 678.4 *m/z*, the same as obtained by **N1** fragmentation (data not shown). This pointed out that the phosphate group on the proximal glucosamine was not stoichiometric. The lack of the phosphate group was a consequence of the 5% acetic acid hydrolysis. In fact, in a recently published paper [18], where the oligosaccharidic portion of the LPS from *Salinivibrio sharmensis* was characterized after mild and strong alkaline treatment of the LPS, the phopshorylation was stoichiometric. On the basis of all the data obtained from the mass spectra as well as from the chemical analysis, the structure of the lipid A from the *S. sharmensis* strain BAG^T^ is depicted in Scheme 1.

## 3. Experimental Section

### 3.1. Isolation of the LPS

*S. sharmensis* strain BAG^T^ (=ATCC BAA-1319^T^ = DSM 18182^T^), isolated from samples collected in a small permanent saline lake in Ras Mohammed Park, located at Sharm el-Sheikh (Egypt) [13], was grown at 35 °C and at pH 9.0, using a medium containing the following components (g/L): yeast extract 10.0, NaCl 100.0, Na_3_-citrate 3.0, Na_2_CO_3_ 3.0, KCl 2.0, MgSO_4_·7H_2_O 1.0, MnCl_2_·4H_2_O 0.00036, FeSO_4_ 0.05 (NaCl and Na_2_CO_3_ were autoclaved separately). The medium was autoclaved for 20 min at 121 °C. The inoculum was prepared using a dilution of 1:50 v/v. Bacterial growth was directly monitored in an UV/Vis spectrophotometer DU 730 (Beckman Coulter) by utilizing the change in optical density at 540 nm. After incubation for 24 h at an absorbance of 2.0, the cells were collected by centrifugation at 10,000× *g* and lyophilized. Dried bacteria cells (7 g) were extracted by PCP method [14], obtaining 103 mg of LPS (yield 1.5% of dried cells).

### 3.2. Mild Acid Hydrolysis of the LPS

The LPS (30 mg) was incubated with 5% aqueous CH_3_COOH (3 mL) for 4 h at 100 °C. The sample was then centrifuged at 10,000× *g* for 15 min and two fractions were recovered: the lipid A (5.3 mg) and the saccharidic portion (22.7 mg).

### 3.3. Chemical Analysis

A sample of the lipid A fraction (0.5 mg) was dried and methanolyzed, as already described elsewhere [12]. Briefly, 1 mL of 1 M HCl/CH_3_OH was added to the sample and the reaction was carried out at 80 °C for 20 h. The crude mixture was extracted twice with hexane. The methanol layer, containing the methyl glycosides, was dried and the residue acetylated by treating with pyridine (200 μL) and acetic anhydride (100 μL) at 100 °C for 30 min. Then both the hexane layer, containing the fatty acids as methyl esters derivatives, and the acetylated methyl glycosides were analyzed by GC-MS. The analyses were performed on a Agilent Technologies gas chromatograph 6850A equipped with a mass selective detector 5973 N and a Zebron ZB-5 capillary column (Phenomenex, 30 m × 0.25 mm i.d., flow rate 1 mL/min, He as carrier gas). The following temperature program was used for lipid analysis—140 °C for 3 min, 140 °C→280 °C at 10 °C/min. Acetylated methyl glycosides were analyzed with the following temperature programs—150 °C for 3 min, 150 °C→240 °C at 3 °C/min.

### 3.4. NH_4_OH Hydrolysis of Lipid A

Lipid A (0.6 mg) was incubated with conc. NH_4_OH (100 μL) as reported [17]. The sample was dried and analyzed by ESI FT-ICR mass spectrometry.

### 3.5. ESI FT-ICR Mass Spectrometry

Electrospray ionization Fourier transform ion cyclotron (ESI FT-ICR) mass spectrometry was performed in the negative and positive ion mode using an APEX QE (Bruker Daltonics) equipped with a 7 Tesla actively shielded magnet. For the negative-ion spectra, samples (~10 µg) were dissolved in a 50:50:0.001 (v/v/v) mixture of 2-propanol, water, and triethylamine (pH 8.5). The samples were sprayed at a flow rate of 2 µL min^−1^. Capillary entrance voltage was set to 3.8 kV and drying gas temperature to 200 °C. The instrument was externally calibrated with appropriate standards. The negative ion mass spectra were charge deconvoluted, and the mass numbers given refer to the monoisotopic mass of the neutral molecules.

The MS/MS lipid species were analyzed in the positive ion mode using a 50:50:0.03 (v/v/v) mixture of 2-propanol, water, and 30 mM ammonium acetate (pH 4.5). Small amounts of triethylamine were added to generate triethylamine adduct ions, which are favorable as precursor ions for MS/MS [16]. Precursor ions were isolated in the ICR-cell and then fragmented by infrared multiphoton dissociation (IRMPD). For this, the unfocused beam of a 35-watt, CO_2_ laser (Synrad) was directed through the center of the trap. The duration of laser irradiation was adapted for optimal fragmentation and varied between 50 and 200 ms, and fragment ions were detected after a delay of 0.5 ms.

## 4. Conclusions

In the organization of the microbial membrane, lipopolysaccharides occupy the outer leaflet, and therefore they are the first to come in contact with the external habitat. This is the reason why they are thought to play a key role in the adaptation mechanisms of extremophilic microorganisms. In particular, the nature and distribution of fatty acids belonging to membrane phospholipids and lipid A may change according to the external environment. 

The peculiar acylation of the lipid A of the haloalkalophilic *Salinivibrio sharmensis*, ranging from di- to hexa-acyl structures, might be connected with the supramolecular structure of its membrane which helps the bacterium to survive in an extreme habitat such as a saline lake.

Depending on the nature and distribution of fatty acyl chains, lipid A may induce a pro-inflammatory or an anti-inflammatory response after interaction with host cells receptors. In particular, lipid A molecules isolated from extremophilic non-pathogenic microorganisms potentially have interesting biological activities and their structure elucidation is the mandatory starting point for the understanding of both adaptation and host-bacterium recognition mechanisms.

In this work we established the structure of *S. sharmensis* lipid A. It is composed of a family of molecules with different acylation patterns; the fatty acid C14:0(3-OH) is always found to be acyloxyamide linked to both glucosamines of the saccharidic backbone, while position 3 and 3′ are substituted with C12:0(3-OH). The distribution of secondary acyl chains in the hexa-acyl species is asymmetric (4 + 2), since C12:0 and C14:0 are both linked as acyloxyacyl to the primary fatty acids of the distal glucosamine. In addition C14:0 can be replaced by C16:1 or C12:0. The overall fatty acid distribution resembles that of *E. coli* lipid A [19], even though the chain length of primary *O*-linked fatty acids is different. On the contrary *Neisseria meningitidis* lipid A [20] shows the same distribution of primary fatty acids, but its hexa-acylated lipid A species displays an overall symmetric 3 + 3 fatty acids distribution, since C12:0 secondary fatty acids are linked as acyloxyacyl at position 2 and 2′, respectively. *N. meningitidis* lipid A, is not toxic and shows little agonist behavior [21], it is a good candidate to be used as vaccine adjuvant. On this basis the biological activity of the lipid A from *Salinivibrio sharmensis* is also worth being investigated. 
